# Rit2 silencing in dopamine neurons drives a progressive Parkinsonian phenotype

**DOI:** 10.21203/rs.3.rs-2944614/v1

**Published:** 2023-05-25

**Authors:** Patrick J. Kearney, Yuanxi Zhang, Yanglan Tan, Elizabeth Kahuno, Tucker L. Conklin, Rita R. Fagan, Rebecca G. Pavchinskiy, Scott A. Shafer, Zhenyu Yue, Haley E. Melikian

**Affiliations:** 1 Brudnick Neuropsychiatric Research Institute, Department of Neurobiology, UMASS Chan Medical School, Worcester, MA; 2 Graduate Program in Neuroscience, Morningside Graduate School of Biomedical Sciences, UMass Chan Medical School, Worcester, MA; 3 Mass Spectrometry Facility, University of Massachusetts Medical School, Shrewsbury, MA; Department of Biochemistry and Molecular Pharmacology, UMASS Chan Medical School, Worcester, MA; 4 Department of Neurology and Friedman Brain Institute, Icahn School of Medicine at Mount Sinai, New York, NY.

## Abstract

Parkinson’s disease (PD) is the second most prevalent neurodegenerative disease and arises from dopamine (DA) neuron death selectively in the substantia nigra pars compacta (SNc). Rit2 is a reported PD risk allele, and recent single cell transcriptomic studies identified a major RIT2 cluster in PD DA neurons, potentially linking Rit2 expression anomalies to a PD patient cohort. However, it is still unknown whether Rit2 loss itself is causative for PD or PD-like symptoms. Here we report that conditional Rit2 silencing in mouse DA neurons drove a progressive motor dysfunction that was more rapid in males than females and was rescued at early stages by either inhibiting the DA transporter (DAT) or with L-DOPA treatment. Motor dysfunction was accompanied by decreases in DA release, striatal DA content, phenotypic DAergic markers, and a loss of DA neurons, with increased pSer129-alpha synuclein expression. These results provide the first evidence that Rit2 loss is causal for SNc cell death and a PD-like phenotype, and reveal key sex-specific differences in the response to Rit2 loss.

## Introduction

Parkinson’s disease (PD) is a complex, progressive, neurodegenerative disorder characterized by SNc DA neuron (DAN) death(1,2). PD prevalence is higher in males and PD phenotypes often go unnoticed until >75% of SNc neurons have died(3,4). Phenotypically, PD patients exhibit profound motor impairment that includes bradykinesia, resting tremor, muscular rigidity, lack of coordination, and postural instability(5). These symptoms are due to SNc DAN cell death and concomitant diminished striatal DA signaling, and PD therapeutic strategies typically aim to boost DA production in the remaining DAN population(6).

Rit2 (AKA: Rin, **R**as-like **i**n **n**eurons) is a small, neuronal, ras-like GTPase with enriched expression in SNc DANs(7). Rit2 directly interacts with the DA transporter (DAT), and is required for regulated DAT membrane trafficking(8–10). In cultured cell models, Rit2 is required for EGF- and NGF-mediated neurite outgrowth, NGF-mediated ERK phosphorylation, and cell viability(11–14). Genome-wide association studies (GWAS) link Rit2 genetic anomalies to PD(15–26), as well as to other neuropsychiatric disorders including essential tremor(24), schizophrenia(24,27), autism spectrum disorder(28,29), bipolar disorder(24), and speech delay(30). Significant Rit2 mRNA decreases were reported in postmortem PD patient SNc(31), and a major transcriptomic cluster was recently identified in SNc DANs from PD subjects that exhibits striking Rit2 expression loss(32). Moreover, Rit2 overexpression is sufficient to rescue cellular and behavioral deficits in an α-synuclein α-syn) mouse PD model(33). Together, these findings suggest that Rit2 plays a key role in DAN function and viability, and may be a critical factor in PD pathogenesis. However, it is still completely unknown whether Rit2 loss is, itself, causative for DAN degeneration and/or PD symptomology. Here, we leveraged our previously described approach for conditional Rit2 silencing in mouse DANs(8,9,34) to directly test this possibility. Our results point to a direct role for Rit2 in DA neuron viability, and that Rit2 loss results in a progressive PD-like phenotype with sex-specific differences in males and females.

## Results

### Rit2 silencing leads to sex-specific, progressive motor dysfunction

We previously leveraged the TET-off approach to conditionally silence Rit2 in Pitx3^*IRES-tTA*^ midbrain DANs(8,9,34), and reported that DAergic Rit2 silencing in both VTA and SNc for 4–6 weeks had no significant effect on either male or female baseline locomotion in the open field(34). Given that PD is typically late onset and progressive, we aimed to more comprehensively investigate the impact of conditional Rit2 knockdown (KD) in DANs on more complex motor behaviors at either short-term (4–6 weeks; ST) or long-term (5–6 months; LT) timepoints in both male and female mice. Pitx3^*IRES-tTA*^ mouse VTA were bilaterally injected with AAV9-TRE-shRit2, which we previously reported drives shRit2 expression selectively in DANs in both VTA and SNc, due to AAV9 spread(9,34). Consistent with our previous reports, AAV9TRE-shRit2 significantly decreased Rit2 mRNA in both ST and LT male and female *Pitx3*^*IRES-tTA*^ mouse midbrains, as compared to AAV9-TRE-eGFP injected controls (Supplemental Figure 1AD). We previously reported that ST Rit2 KD had no effect on baseline locomotion. Similar to our previous finding in ST shRit2 mice, LT Rit2 KD likewise did not significantly affect horizontal locomotion in either male or female mice (Supplemental Figure 1E,F), nor was their fine movement significantly affected (Supplemental Figure 1G,I). There was additionally no change in male vertical motion (Supplemental Figure 1H), whereas female mice exhibited significantly increased vertical locomotion (Supplemental Figure 1J).

**Figure 1.**
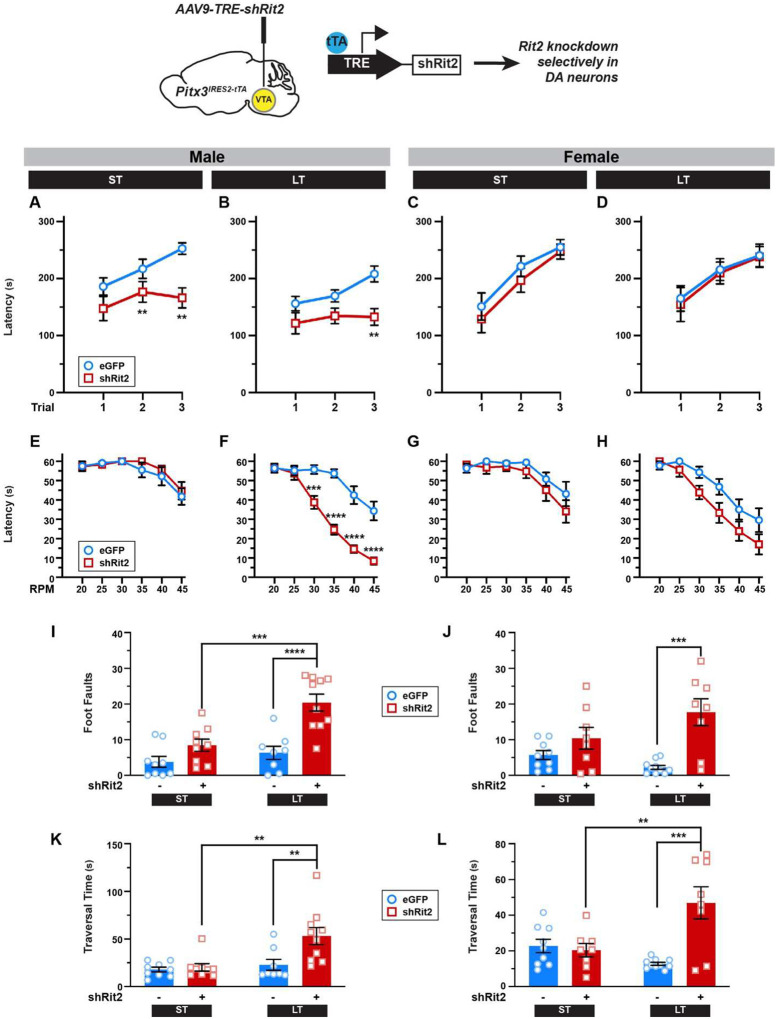
Conditional Rit2 silencing in DA neurons leads to progressive, but differential, motor dysfunction in males and females. *Pitx3*^*IRES-tTA*^ mouse midbrains were bilaterally injected with either AAV9-TRE-eGFP or -shRit2 and mice were assessed either 4–5 weeks (ST) or 25 weeks (LT) post-injection. Data were analyzed by two-way repeat measures ANOVA with Sidak’s multiple comparison test (A-H), or two-way ANOVA with Tukey’s multiple comparison test (I-L)*. Top: Experimental schematic. Pitx3*^*IRES-tTA*^ mouse midbrains were bilaterally injected with either AAV9-TRE-eGFP or -shRit2 and mice were assessed either 4–5 weeks (ST) or 25 weeks (LT) post-injection. **(A-D)** *Accelerating Rotarod:* Mice were assessed over 3 consecutive trials as described in Methods. Rit2 silencing in DA neurons abolished motor learning in male ST (**A**. Trial: p=0.001, F_(2, 41)_= 8.95; Virus: p=0.002, F_(1, 24)_ = 11.69; trial × virus: p=0.03; *p<0.005; n=9–11) and LT (**B**. Trial: p=0.01, F_(2, 34)_=5.22; Virus: p=0.02, F_(1, 17)_=7.02; **p=0.005, n=8–11) mice, but had no effect on ST (**C**. Trial: p=0.002, F_(2, 48)_=6.82; Virus: p=0.72, F_(1, 48)_=0.12; n=1112) or LT (**D**. Trial: p=0.002, F_(2, 48)_=6.82, Virus: p=0.72, F_(1,48)_=0.12, n=8–10) female motor learning. **(E-H)** *Fixed-speed Rotarod:* Mice were assessed over the indicated consecutive speeds as described in Methods. **E,F**. Rit2 silencing did not affect ST males (**E**. Rate: p<0.0001, F_(5,70)_=11.59, Virus: p=0.48, F_(1,14)_=0.53, n=8), but significantly diminished LT male rotarod performance (**F**. Rate: p<0.0001, F_(5,102)_=46.88, Virus: p<0.0001, F_(1,102)_=98.31, Rate × Virus: p<0.0001, F_(5,102)_=5.34; ***p<0.001, ****p<0.0001, n=8–11) **G,H**. Rit2 silencing had no effect on female rotarod performance at the ST timepoint (**G**. RPM: p<0.0001, F_(2, 35)_=12.79, Virus: p=0.22, F_(1,15)_=1.637; n=8) but significantly diminished performance at the LT timepoint (**H**. RPM: p<0.0001, F_(5,108)_=6.29; Virus: p=0.006, F_(1,108)_=12.48; n=10). **I-L**. *Challenge balance beam:* Mice were assessed on the challenge balance beam as described in Methods. Mean foot fault numbers **(I,J)** and beam traversal times (seconds) **(K,L)** were analyzed. Rit2 silencing had no effect on ST male foot faults, but increased LT male foot faults (**I**. Time: p=0.0007, F_(1,32)_=14.23; Virus: p<0.0001, F_(1, 32)_=23.83; Time × virus: p=0.02, F_(1, 32)_=6.01; ***p=0.004, ****p<0.0001, n=8–10) and traversal times (**K**. Time: p=0.004, F_(1,32)_=9.52; Virus: p=0.01, F_(1, 32)_=7.02; Time × virus: p=0.03, F_(1, 32)_=5.17; **p<0.01, n=8–10). Rit2 silencing had no effect on ST female foot faults, but increased LT female foot faults (**J**. Time: p=0.42, F_(1,31)_=0.68; Virus: p=0.0002, F_(1,_ 31)=18.59; Time × virus: p=0.03, F_(1, 31)_=5.36; ***p=0.0003, n=8–10) and traversal times (**L**. Time: p=0.10, F_(1,31)_=2.85; Virus: p=0.003, F_(1, 31)_=10.64; Time × virus: p=0.0008, F_(1, 31)_=13.95; **p=0.005, ***p=0.0001, n=8–10).

We next asked whether either ST or LT DAergic Rit2 KD impacted more complex motor behaviors, such as motor learning and coordination, assessed on rotarod and challenge balance beam assays. In male mice, both ST and LT DAergic Rit2 KD significantly decreased performance on the accelerating rotarod compared to controls (Figure 1A,B), whereas female mouse rotarod performance was not significantly affected by either ST or LT DAergic Rit2 KD (Figure 1C,D). Accelerating rotarod deficits could be due to either learning or coordination deficits. To discriminate between these possibilities, we assessed performance on the fixed-speed rotarod and challenge balance beam. Despite their accelerating rotarod deficits, male ST shRit2 mice did not exhibit any significant deficits on either the fixed-speed rotarod (Figure 1E), or challenge balance beam (Figure 1I,K), as compared to controls. Similar to males, female ST shRit2 mice did not exhibit any significant deficits on either the fixed-speed rotarod (Figure 1G) or challenge balance beam (Figure 1J,L), as compared to controls. We further assessed both mouse gait and grip strength following ST Rit2 KD, and found no differences between shRit2 mice and controls, in either males or females (Supplemental Figure 2).

Given that ST Rit2 KD only affected male accelerating rotarod performance, we next asked whether longer Rit2 silencing would lead to progressive motor dysfunction. Following LT Rit2 KD, males continued to exhibit significantly poorer performance than controls on the accelerating rotarod (Figure 1B), and females still exhibited no significant effect on accelerating rotarod performance (Figure 1D). However, LT Rit2 KD drove a significant deficit in fixed-rotarod performance in both males (Figure 1F) and females (Figure 1H), and both foot faults and beam traversal times were significantly increased in both male (Figure 1I,K) and female (Figure 1J,L) LT shRit2 mice on the balance beam, as compared to both control and ST Rit2 KD mice. Gait analysis revealed that multiple gait parameters were unaffected by ST Rit2 KD in both males (Supplemental Figure 2A,E,I,M) and females (Supplemental Figure 2C,G,K,O). However, there was a differential impact on gait in males and females following LT Rit2 KD. LT shRit2 males completed significantly fewer gait analysis trials (Supplemental Figure 2B) and had significantly narrower forelimb stride widths (Supplemental Figure 2J), whereas LT shRit2 females had significantly wider hindlimb stride widths (Supplemental Figure 2L). Despite the observed coordination and gait deficits, all LT shRit2 mice also had significantly increased four-limb grip strength (Supplemental Figure 2R,T). Taken together, the behavioral data suggests that DAergic Rit2 is specifically required for male motor learning and that prolonged Rit2 suppression leads to progressive motor coordination and gait deficits in both males and females.

### Short-term DAergic Rit2 silencing blunts DA release in males

**Figure 2.**
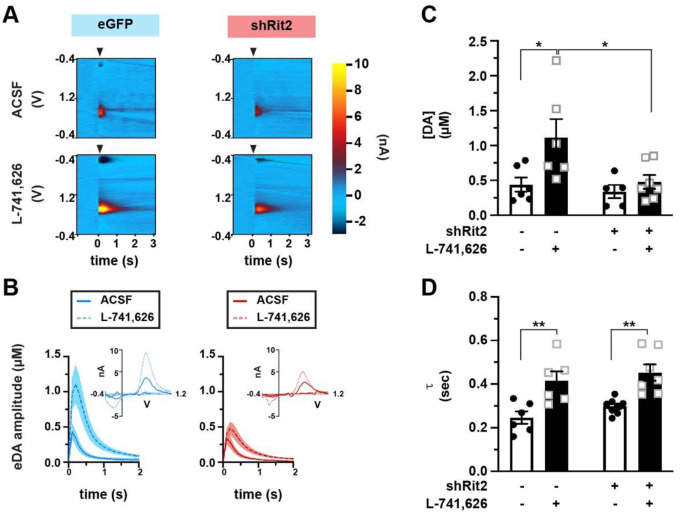
Short-term DAergic Rit2 silencing impacts DA release, but not clearance in males. *Ex vivo fast-scan cyclic voltammetry: Pitx3*^*IRES-tTA*^ mouse VTA were bilaterally injected with either AAV9-TRE-eGFP (n=6) or AAV9-TRE-shRit2 (n=5–7) and electrically evoked DA transients were measured *ex vivo* in acute dorsal striatum as described in Methods. Values were analyzed by two-way ANOVA with Tukey’s multiple comparison test. **(A)** *Representative voltammograms:* Voltammograms displaying evoked current over voltage cycles and time, in slices from eGFP- and shRit2-injected mice, recorded in either ACSF or 25nM L-741,626, as indicated. Arrowheads indicate delivery of single, squared wave pulse. **(B)** *Dopamine transients:* Representative evoked DA transients in slices in slices from eGFP- and shRit2-injected mice, recorded in either ACSF or 25nM L-741,626, ±S.E.M. (shaded areas), as indicated. **(C)** *Average amplitudes:* DA transient amplitudes are presented in μM ±S.E.M. Virus: p=0.03, F_(1, 20)_=2.83; Drug: p=0.02, F_(1, 20)_=6.58; *p<0.05. **(D)** Average decay tau, presented in seconds ±S.E.M. Virus: p=0.16, F_(1, 23)_=2.08; Drug: p<0.0001, F_(1, 23)_=27.43; **p<0.01.

Motor learning deficits in males in response to ST DAergic Rit2 silencing could be due to altered DA release. To test this possibility, we leveraged fast-scan cyclic voltammetry (FSCV) to measure both DA release and clearance in *ex vivo* dorsal striatal slices (Figure 2A,B). Given the viability issues inherent to acute brain slices prepared from older animals, we limited our FSCV studies to ST shRit2- and control-injected males, in which we observed a motor learning deficit. We(9) and others(35) previously reported that DRD2 autoreceptors significantly blunt DA transient amplitudes and accelerate DA clearance. Indeed, in control mice DA transient amplitudes were significantly smaller when evoked in ACSF as compared to those evoked in the presence of L-741,626 (25nM, Figure 2C), a DRD2-specific antagonist, as we previously reported(9). In shRit2 mice, DA transient amplitudes recorded in ACSF were not significantly different transients from control mice (Figure 2C). However, unlike control DA transients, DA amplitudes recorded in the presence of L-741,626 were not significantly greater than those recorded in ACSF, and were significantly smaller than amplitudes recorded in L-741,626 from control mice. Moreover, we previously reported that ST Rit2 silencing in males decreased DAT surface levels by ~50% in dorsal striatum (DS). Despite this reduction in DAT, Rit2 silencing did not significantly affect DA clearance times (Figure 2D), and we still observed DRD2-mediated enhancement of DA clearance in slices from both control and shRit2 mice, suggesting that DRD2 signaling was intact (Figure 2D). Taken together, these results suggest that males locomotor deficits following ST Rit2 silencing may be, in part, due aberrant DA signaling.

### Rit2 silencing suppresses the DAergic phenotype with earlier manifestation in males than females

**Figure 3.**
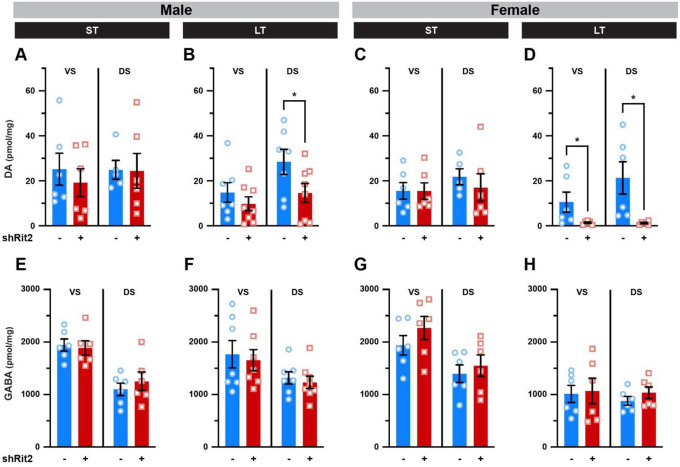
Long-term, but not short-term, Rit2 silencing decreases striatal DA content. Mass spectrometry. Dorsal (DS) and ventral (VS) striata were dissected from male and female control and shRit2 mice at the indicated timepoints, and total DA and GABA content were measured by LC/MS/MS as described in Methods. Data are presented as pmol of the indicated neurotransmitter per mg tissue, and were analyzed by unpaired, one-tailed (DA) or two-tailed (GABA) Student’s t test. **(A-D)** *Striatal DA Content*: ST Rit2 silencing had no effect on DA content in male VS and DS (**A**. VS: p=0.27; DS: p=0.48, n=5–6), whereas LT Rit2 silencing decreased DA in DS, but not VS (**B**. VS: p=0.18; DS: p=0.03, n=7–8). ST Rit2 silencing had no effect on DA content in female VS and DS (**C**. VS: p=0.49; DS: p=0.26, n=5–6), but significantly decreased DA in LT females in both VS and DS (**D**. VS: *p=0.04; DS: *p=0.02, t test with Welch’s correction, n=6). **(E-H)** *Striatal GABA content*. Rit2 silencing had no effect on GABA content in either VS or DS in ST males (**E**. VS: p=0.75; DS: p=0.50, n=6), LT males (**F**. VS: p=0.74; DS: p=0.61, n=78), ST females (**G**. VS: p=0.28; DS: p=0.58, n=6), or LT females (**H**. VS: p=0.86; DS: p=0.29, n=6).

Given our FSCV results, we hypothesized that motor deficits observed in ST males, and in both males and females following LT Rit2 silencing, could potentially be due to a loss in DAergic tone. To test this possibility, we first measured striatal DA content using mass spectroscopy in male and female dorsal (DS) and ventral (VS) striata following ST and LT Rit2 silencing. In ST shRit2 mice, total DA content was not significantly affected in either DS or VS from either male or female mice as compared to their respective controls (Figure 3A,C). However, DA content was significantly reduced in LT shRit2 male DS (Figure 3B) and LT shRit2 female VS and DS (Figure 3D) as compared to controls. Importantly, total striatal GABA content was not altered in male or female VS or DS at any timepoint (Figure 3E–H), demonstrating specific changes in DANs and not global changes in striatal neurotransmitter content.

**Figure 4.**
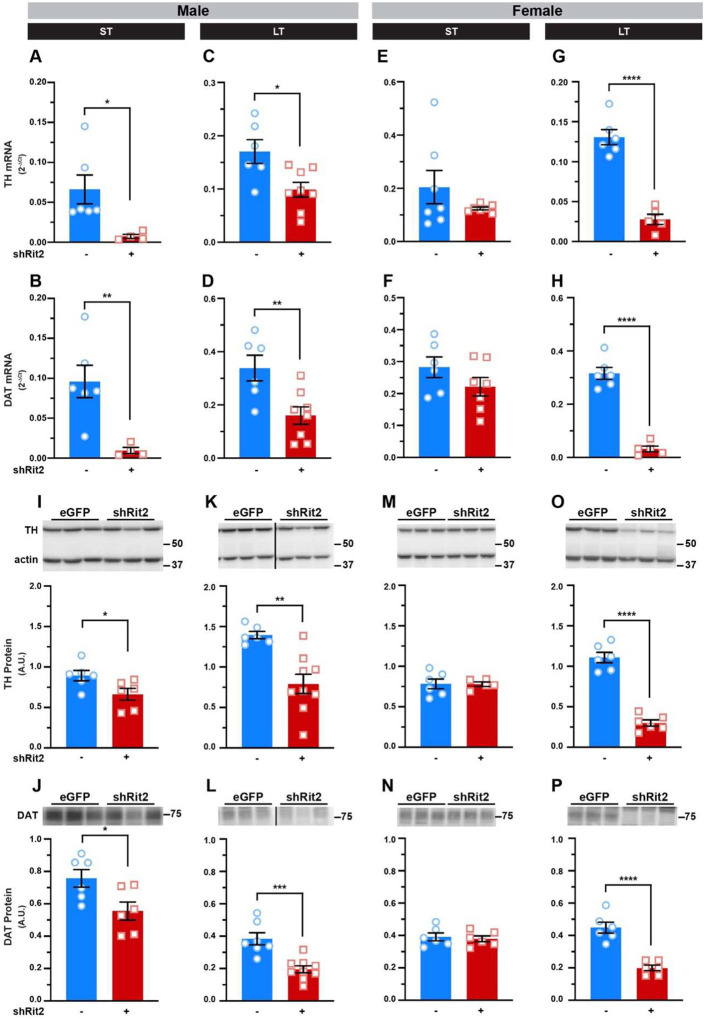
DAergic Rit2 silencing diminishes DAergic mRNA and protein expression. Ventral midbrain and striatum were dissected from male and female control and shRit2 mice at the indicated timepoints, and TH and DAT mRNA and protein were measured in ventral midbrain and striatal subregions, respectively, as described in Methods. Data were analyzed by unpaired, two-tailed Student’s t test. (**A-D**) Male ventral midbrain RT-qPCR. TH (**A**. *p=0.02 with Welch’s correction, n=4–7) and DAT (**B**. **p=0.008 with Welch’s correction, n=4–6) mRNA were significantly decreased in ST shRit2 VM and both TH (**C**. *p=0.01, n=6–8) and DAT (**D**. **p=0.008, n=6–8) remained suppressed in LT shRit2 ventral midbrain as compared to eGFP controls. (**C-F)** Female ventral midbrain RT-pPCR. Neither TH (**E**. p=0.25 with Welch’s correction, n=7) nor DAT (**F**. p=0.19, n=6–7) mRNA were significantly affected in ST shRit2 ventral midbrain, whereas both TH (**C**. ****p<0.0001, n=5–6) and DAT (**D**. ****p<0.0001, n=5–6) were diminished in LT shRit2 ventral midbrain as compared to eGFP controls. (**I-L)** Male striatal protein. Top: Representative striatal immunoblots for each protein, showing 3 independent mouse lysates each for control and shRit2 mice. Molecular weight markers are indicated in kDa. TH (**I**. *p=0.04, n=6) and DAT (**J**. *p=0.03, n=6) protein levels were significantly decreased in ST shRit2 striata, and both TH (**K**. ***p=0.0008 with Welch’s correction, n=6–9) and DAT (**L**. ***p=0.0004, n=7–9) continued to be significantly decreased in LT shRit2 striata. (**M-P**) Female striatal protein. Top: Representative striatal immunoblots for each protein, showing 3 independent mouse lysates each for control (eGFP) and shRit2 mice. Neither TH (**M**. p=0.96) nor DAT (**N**. p=0.71, n=5–6) striatal protein levels were significantly diminished in shRit2 striata as compared to controls. In LT shRit2 striata, both TH (**O**. ****p<0.0001, n=6) and DAT (**P**. ****p<0.0001, n=6) protein levels were significantly reduced as compared to controls.

Given Rit2’s association with PD and the profound changes motor function and DAergic tone observed with LT Rit2 silencing, we asked whether Rit2 silencing impacted DAN viability. We first assessed DAergic gene and protein expression in isolated ventral midbrain (vMB) and striatum, respectively, following ST and LT Rit2 silencing. In males, RT-qPCR studies revealed that ST Rit2 KD significantly decreased tyrosine hydroxylase (TH) and DAT mRNA in vMB (Figure 4A,B), and quantitative immunoblotting revealed that striatal TH and DAT protein were also significantly reduced (Figure 4I,J). Unsurprisingly, following LT Rit2 KD, male TH and DAT vMB mRNA (Figure 4C,D) and striatal TH and DAT protein (Figure 4K,L) remained significantly diminished compared to controls. In females, TH and DAT mRNA (Figure 4E,F) and protein (Figure 4M,N) were unaffected following ST Rit2 KD. However, following LT Rit2 silencing females exhibited robust and significant loss in vMB TH and DAT mRNA (Figure 4G,H), as well as striatal TH and DAT protein (Figure 4O,P).

We further asked whether Rit2 silencing impacted TH activation, by measuring pSer40-TH via immunoblot. When normalized to actin, pSer40-TH was significantly reduced in ST shRit2 male striatum (Supplemental Figure 3A) and trended to decrease in LT shRit2 males (Supplemental Figure 3B). However, proportion of pSer40-TH to total TH was not significantly different in ST or LT shRit2 male mice as compared to controls (Supplemental Figure 3E,F), suggesting that functional regulation of the TH pool is intact. In females, ST Rit2 silencing had no effect on pSer40-TH or the fraction of pSer40-TH (Supplemental Figure 3C,G), however we detected a drastic loss pSer40-TH loss following LT Rit2 silencing in females (Supplemental Figure 3D), as well as the fraction of pSer40-TH (Supplemental Figure 3H), suggesting the TH is dysregulated in this population.

Given the profound losses in TH and DAT expression, as well as DA content, we further asked whether other characteristic ventral midbrain DAergic mRNAs were affected by shRit2. In both ST and LT shRit2 males, DRD2 and Pitx3 mRNA were significantly decreased (Supplemental Figure 4A,B,E,F), and Nurr1 was significantly diminished following LT, but not ST, Rit2 silencing (Supplemental Figure 4I,J). In females, ST Rit2 silencing did not significantly affect DRD2, Pitx3, or Nurr1 mRNA levels (Supplemental Figure 4C,G,K). However, by the LT timepoint all three DAergic markers were significantly diminished (Supplemental Figure 4D,H,L). Taken together these data demonstrate that DAergic Rit2 silencing results in downregulation of all DAergic genes consistent with the progressive loss in DAergic tone.

We additionally tested whether gene silencing in response to Rit2 KD was specific to DAergic genes or whether pan neuronal and/or ubiquitous genes are also affected by Rit2 silencing (Supplemental Figure 5). We measured vMB expression of the ubiquitously expressed Rit2 homolog, Rit1, and Vps35, a core retromer component that is also associated with PD. Surprisingly, both Rit1 and Vps35 gene expression were increased in ST, but not LT shRit2 males (Supplemental Figure 5A,B). Rit1 and Vps35 expression were unaffected in ST shRit2 females (Supplemental Figure 5C,G) but significantly increased at the LT timepoint (Supplemental Figure 5D,H).

### Long term Rit2 silencing results in DAN degeneration

**Figure 5.**
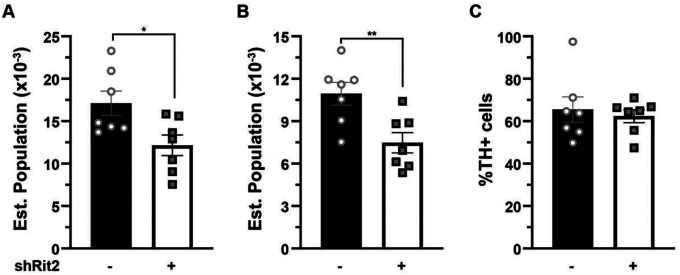
Prolonged DAergic Rit2 silencing decreases the substantia nigra DA neuron population. *Stereological analysis of SNc neurons*. Pitx3^IRES-tTA^ males and female mouse VTA were bilaterally injected with either AAV9-TRE-eGFP or AAV9-TRE-shRit2 and brains were fixed, sectioned and stained with cresyl violet and TH-specific antibodies at the LT timepoint. Total and TH+ cell numbers were counted as described in Methods. LT male and female data were pooled. LT Rit2 KD significantly decreased total Nissl+ (A. *p=0.02) and TH+ (**B**. **p=0.008) cells, but did not significantly affect the %neurons that were TH+ (**C**. p=0.64) in SNc. Unpaired, two-tailed Student’s t test, n=7.

Loss of the DAergic phenotype may be due to cell death, or quiescence of the DAergic phenotype without cell death. To discriminate between these possibilities, we performed stereological counting to measure the number of total (Nissl stain) and dopaminergic (TH+) SNc neurons following at the LT Rit2 KD timepoint, where both males and females exhibit significant motor dysfunction. LT Rit2 KD significantly decreased total Nissl+ cells in males and females, combined (Figure 5A), as well as TH+ neurons (Figure 5B), consistent with cell death. There was, however, no difference in the proportion of neurons that were TH+ in LT Rit2 KD as compared to controls (Figure 5C), suggesting that with neuronal losses occurred primarily in the DAergic population.

### Prolonged DAergic Rit2 silencing increases modified α-synuclein levels in the striatum

**Figure 6.**
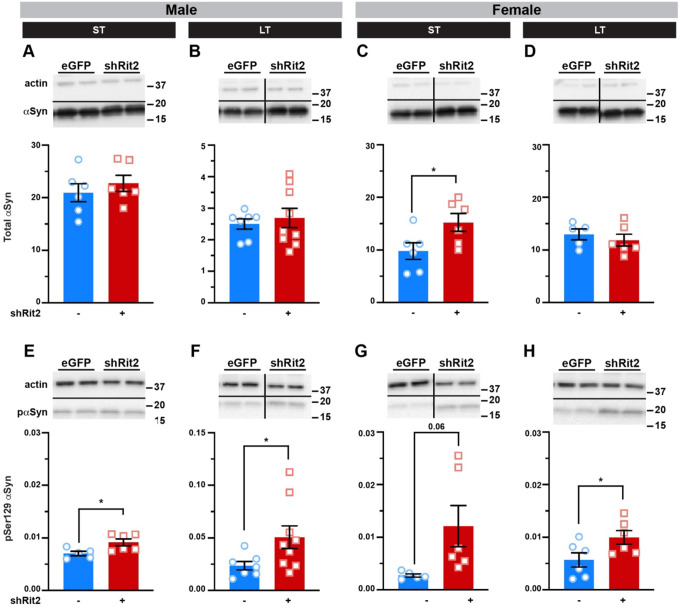
Prolonged DAergic Rit2 silencing increases PD-associated protein biomarkers in striatum. *Quantitative Immunoblotting*. Pitx3^IRES-tTA^ mouse VTA were bilaterally injected with either AAV9-TRE-eGFP or AAV9-TRE-shRit2. Striatal lysates were collected 4–5 weeks (ST) or 5–6mo (LT) post-injection and αSyn and αS129-aSyn levels were measured by quantitative immunoblot, normalized to actin, as described in Methods. *Tops:* Representative immunoblots showing two independent mouse lysates per virus. **(A-D)** *αSyn levels:* shRit2 had no effect on total αSyn levels in males at either ST (**A**. p=0.46, n=6) or LT (**B**. p=0.62, n=7–9) timepoints. ST shRit2 significantly increased αSyn in females (**C**. *p=0.04, n=6), but had no effect in LT females as compared to controls (**D**. p=0.50, n=6). (**E-H)** pS129-*αSyn levels*: pS129-αSyn was significantly increased by shRit2 in ST (E. *p=0.02, n=6) and LT (**F**. *p=0.04, n=7–9) males. pS129-αSyn trended towards a significant increase by shRit2 in ST females (**G**. p=0.06, n=6) and was significantly increased in LT females (**H**. *p=0.04, n=7–9). Two-tailed, unpaired, Student’s t test.

Given Rit2’s association with PD, and the progressive motor dysfunction we observed following Rit2 silencing, we next asked whether Rit2 silencing was accompanied by a change in α-synuclein, a major component of Lewy bodies, which are the hallmark of PD neuropathology (ref). To test this, we probed striatal lysates from all mouse cohorts for αSyn and pSer129-αSyn, which is markedly increased in idiopathic PD(36). Total αSyn levels were not significantly affected in either ST or LT shRit2 males (Figure 6A,B) nor in LT females (Figure 6D), but were significantly increased in ST shRit2 females (Figure 6C). Importantly, shRit2 drove a significant increase in pSer129-αSyn in ST and LT shRit2 males (Figure 6E,F) and in LT females (Figure 6H), and strongly trended for an increase in ST females (Figure 6F). Together, these data indicates that in addition to profound motor deficits, and DAN degeneration, LT DAergic Rit2 silencing drives an increase in modified α-synuclein.

### Male motor learning is rescued with Parkinson’s therapeutics.

**Figure 7.**
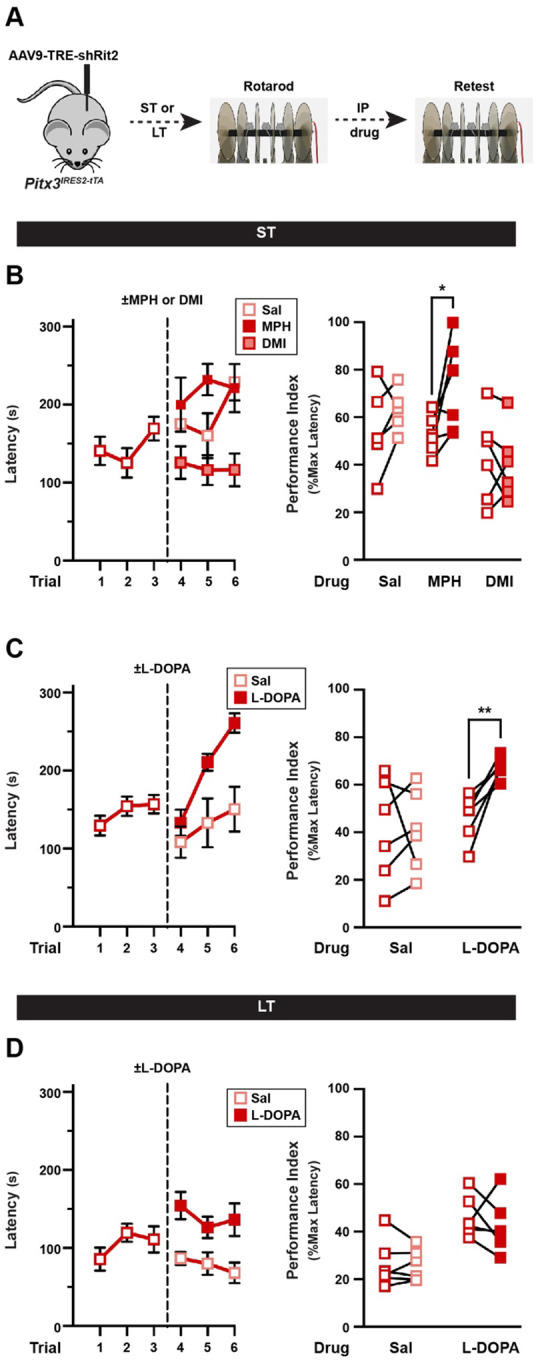
Male motor learning deficits are rescuable with Parkinson’s therapeutics at early, but not late, Rit2 silencing timepoints. *Accelerating rotarod rescue studies*. **(A)** *Experimental schematic*. Pitx3^IRES-tTA^ male mouse VTA were bilaterally injected with AAV9-TRE shRit2 and were assessed on the accelerating rotarod at the indicated timepoints as described in Methods. Mice were initially tested for three trials, injected ±the indicated treatment drugs (I.P), and were retested for an additional three trials. Performance indices for each mouse were calculated pre- and post-test, and performance were assessed with a two-tailed, paired Student’s t test **(B)** *Methylphenidate (MPH) treatment:* ST shRit2 mice received either saline, 5mg/kg MPH, or 0.5mg/kg DMI, and were retested 15 min post injection. *Left:* Raw rotarod results presented as latency to fall during the trial. *Right:* Paired pre- and post-test rotarod performance indices. shRit2 mice treated with MPH performed significantly better than pre-injection (*p=0.03), whereas performance was not enhanced by either saline (p=0.28) or DMI (p=0.60), n=5–6. **(C)** *L-DOPA treatment on ST shRit2 males:* ST shRit2 mice were treated ±20mg/kg L-DOPA and were retested 1 hour post-injection. *Left:* Raw rotarod results. *Right:* Paired pre- and post-test rotarod performance indices. shRit2 mice treated with L DOPA (**p=0.005), but not saline (p=0.98) performed significantly better than pre-injection, n=6–7. **(D)** *L-DOPA treatment on LT shRit2 males:* LT shRit2 mice were treated ±20mg/kg L-DOPA and were retested 1 hour post-injection. *Left:* Raw rotarod results. *Right:* Paired pre- and post-test rotarod performance indices. Neither L-DOPA (p=0.48) nor saline (p=0.82) treatment significantly improved performance as compared to pre injection performance, n=6.

The most widely used treatment strategy for PD is to increase DA availability by providing the DA precursor, L-DOPA. Moreover, recent studies suggest that the DAT inhibitor, methylphenidate (Ritalin), may have therapeutic potential in PD(37,38). We asked whether such pharmacological intervention could rescue the motor deficits observed on the rotarod due to Rit2 silencing. We first tested whether increasing extracellular DA levels by inhibiting DAT with methylphenidate could rescue motor learning. ST shRit2 males were assessed on the accelerating rotarod, injected ±methylphenidate (MPH, 5mg/kg, I.P.), and were reassessed 15 min post-injection (see schematic, Figure 7A). MPH treatment significantly improved rotarod performance as compared to vehicle-injected mice (Figure 7B). MPH (aka Ritalin) is a therapeutic psychostimulant that is equipotent at DAT and the norepinephrine transporter (NET) (39). Therefore, to rule out any adrenergic contributions to motor learning rescue, we tested whether rotarod performance was improved with desipramine (DMI), a NET-specific inhibitor. DMI treatment had no significant effect on shRit2 mouse performance (Figure 7B), suggesting that DAT inhibition was specifically responsible for rescued rotarod performance in ST shRit2 males. L-DOPA is the immediate chemical precursor to DA and has been the prevailing treatment for PD since the 1960s(40). Therefore, we next asked whether treating ST and LT shRit2 mice with L-DOPA could rescue motor learning deficits. Mice were assessed on the accelerating rotarod, injected ± L-DOPA (20mg/kg, I.P.), and reassessed 1-hour post-injection. In ST shRit2 mice, L-DOPA robustly and significantly improved rotarod performance (Figure 7C). However, in LT shRit2 mice, L-DOPA treatment had no effect on rotarod performance (Figure 7D). Taken together, these data demonstrate, that while pharmacological intervention can rescue motor deficits exhibited by ST shRit2 mice, the progressive loss of DAergic tone and DANs caused by LT Rit2 KD drives deficits that are not rescuable by pharmacological means.

## Discussion

Rit2 was identified as a PD risk allele in multiple GWAS and, although critical SNPs have been identified, it was unknown whether decreased Rit2 expression itself is detrimental to DAN function and/or viability (15–26). Rit2 mRNA is one of the more highly downregulated genes in postmortem PD patient substantia nigra (31), and defines a specific transcriptomic cluster in single-cell RNAseq studies from post-mortem patients(32). However, whether that diminished Rit2 levels is causal or consequential for PD progression has yet to be determined. Our results demonstrate that prolonged conditional DAergic Rit2 silencing leads to PD-like phenotypes. We observed that conditional DAergic Rit2 knockdown leads to progressive loss of motor coordination (Figure 1) accompanied by decreased DA release (Figure 2), loss of striatal DA content (Figure 3), decreased DAergic gene and protein expression (Figure 4), decreased DA neuron numbers (Figure 5), and increased pSer129-αSyn (Figure 6). Thus, our results demonstrate that Rit2 loss itself is causal for PD-like phenotypes.

Many of these phenotypes we observed significantly affected males at an earlier timepoint than females, consistent with sex-specific differences in PD prevalence and onset. Interestingly, female performance on the accelerating rotarod was completely resistant to Rit2 loss, even at the LT timepoint, despite significant deficits in gait, fix-speed rotarod, and challenge balance beam, and diminished dopaminergic tone. Motor learning on the accelerating rotarod is DAdependent(41,42), however the observed sexual dimorphism raises the possibility that there may be a strong DA-independent component to motor learning in females. Alternatively, it is possible that distinct, sex-specific SNc DAN subpopulations are vulnerable following Rit2 silencing. Future single cell transcriptomic studies will be necessary to test this possibility directly.

While motor learning deficits are specific to shRit2 male mice, shRit2 coordination deficits are sex-independent and are only apparent with prolonged Rit2 silencing (Figure 1). Coordination deficits were accompanied by modest alterations in shRit2 mouse gait (Supplemental Figure 2). Furthermore, LT male and female mice both exhibited increased four-limb grip strength (Supplemental Figure 2). While grip strength typically decreases with PD progression in patients (43), increased mouse grip strength may also reflect rigidity or bradykinesia present in PD. Indeed, increased grip strength is observed in a 6-OHDA lesion rat model of PD (44). If Rit2 silencing reflects the progressive nature of PD pathology, it is entirely possible that these coordination, gait, and grip strength phenotypes may become more severe with further extended Rit2 silencing.

Surprisingly, despite coordination deficits, conditional Rit2 silencing did not perturb horizontal locomotion, even with prolonged silencing (Supplemental Figure 1). PD is a late onset neurodegenerative disorder and motor symptoms are often not overtly apparent until >75% of SNc DANs have died (4). Indeed, our stereological data suggest that at the LT timepoint (~25 weeks post-injection), there is ~32% loss of TH+ neurons in the SNc (Figure 5). Thus, it is possible that longer Rit2 silencing would lead to even further losses in the TH+ population, and more pronounced baseline motor deficits.

While Rit2 silencing diminished DA neuron numbers, the mechanism(s) downstream of Rit2 loss that lead to decreased DAN viability are unknown. To date, the function of Rit2 in neurons remains poorly defined. Rit2 is required for EGF- and NGF-mediated neurite outgrowth in cell culture models (11–13), and is required for NGF-mediated ERK phosphorylation and cell viability (12,14). Our lab previously reported that Rit2 binds directly to the DAT (10) and is required for both PKC- (8) and mGluR5-mediated (9) DAT internalization in *ex vivo* striatal slices. Indeed, conditional mGluR5 silencing in DANs blocked DAT internalization, increased DAT plasma membrane presentation, and likewise resulted in male inability to perform on the accelerating rotarod(9). Thus, ST shRit2 effects may be due, in part, to DAT dysregulation, while LT shRit2 effects may progressively diminish DAN viability. It is worth noting our previous shRit2 study, in which we found that ST shRit2 differentially modulates acute cocaine locomotor responses based on sex, wherein male shRit2 mice exhibit increased cocaine sensitivity, and females exhibit a loss in cocaine sensitivity (34). Thus, although we only observed motor dysfunction in males following ST Rit2 silencing in our current study, there is clearly still an impact in females that does not manifest as a motor behavior.

Despite the observed decreases in striatal DAT protein in ST shRit2 males (Figure 4J), we did not measure any significant change in DA clearance in parallel FSCV studies (Figure 2D). Interestingly, we instead observed that Rit2 silencing dampened DA release as compared to controls (Figure 2C). Decreased DA release was accompanied by decreases in both pSer40-TH (Supplemental Figure 3A) and DRD2 mRNA (Supplemental Figure 4A), raising the possibility that DA synthesis may be altered due to Rit2 silencing. However, DRD2-mediated regulation of DA clearance remained intact following Rit2 KD (Figure 2D), suggesting that DRD2 dysregulation is not likely to mediate the diminished DA release we observed. Rit2 may play some previously undefined role in DA synthesis and/or release that is independent of DRD2 regulation.

In this study, we assessed vMB expression of five DAergic genes which are critical for DA signaling and gene regulation: TH, DAT, DRD2, Pitx3 and Nurr1. Expression of all of these genes was significantly diminished in male and female LT shRit2 mice and all except Nurr1 were decreased in ST shRit2 males (Supplemental Figure 4). Nurr1 is associated with PD progression and conditional ablation of DAergic Nurr1 results in decreased, DAT and TH expression, reduced striatal DA content and locomotor deficits (45,46). Surprisingly, we saw upregulation in the ubiquitously expressed genes, Rit1 and Vps35 (Supplemental Figure 5). Rit1 is the closest homolog to Rit2 and may be upregulated or stabilized to compensate for Rit2 loss, however, whether they functionally overlap is unknown. We also observed increases in the PD biomarker pSer129-αSyn (Figure 6). Previous studies have demonstrated that pSer129-αSyn accumulates in DAergic nuclei and negatively regulates Nurr1 expression (47). Whether Rit2 directly regulates DAergic gene expression, or whether the observed changes are consequences of viability and cell death will need to be determined.

As discussed above, Rit2 silencing results in hypodopaminergic tone, which likely contributes to observed motor learning and coordination deficits. We used two pharmacological approaches to test whether boosting DA availability could rescue motor dysfunction: DAT inhibition with MPH, and L-DOPA treatment. Both MPH and L-DOPA treatments rescued male accelerating rotarod performance following ST Rit2 KD (Figure 7B,C), but was unable to rescue performance following LT Rit2 KD (Figure 7D). Whether L-DOPA is completely ineffective at LT timepoints or whether, as in PD patients, increasing doses are required to overcome DA depletion remains to be tested.

Our study, for the first time demonstrates that DAergic Rit2 is required for DA neuron viability and DA-dependent motor behaviors. We identified a sex dependent role for Rit2 in motor learning and demonstrated the manifestation of progressive, PD-like, gene expression, DA content, and biomarker phenotypes following prolonged Rit2 silencing. With further evaluation to determine whether Rit2 loss leads to the formation of αSyn inclusions, Rit2 silencing could prove to be a novel mouse model for Parkinson’s disease.

## Methods

### Materials

L-DOPA (3788) and L-741,626 (1003) were from Tocris. Methylphenidate and desipramine were from Sigma. All other reagents were from either Sigma-Aldrich or Fisher Scientific and were of the highest possible grade.

### Mice

*Pitx3*^*IRES-iTA/+*^ mice were continuously backcrossed onto the C57Bl/6J background and were the generous gift of Dr. Huaibin Cai (National Institute on Aging). Mice were maintained on 12hr light/dark cycle (lights on at 0700) at constant temperature and humidity. Food and water were available ad libitum and mice were maintained on standard chow. All studies were conducted in accordance with University of Massachusetts Medical School IACUC Protocol 202100046 (formerly A-1506 H.E.M.).

### AAVs and Stereotaxic Surgeries

#### AAVs:

pscAAV-TRE3g-eGFP and pscAAV-TRE3g-miR33-shRit2-eGFP AAV9 particles were produced as previously described(34) by the University of Massachusetts Medical School Viral Vector Core.

#### Survival Surgeries:

Mice aged 3–4 weeks were anesthetized with I.P. 100mg/kg ketamine (Vedco Inc.) and 10mg/kg xylazine (Akorn Inc). To increase viral spread, mice were administered 20% mannitol (NeogenVet) at least 15min prior to viral delivery(48). Anesthetized mice were prepared and placed in a stereotaxic frame (Stoelting Inc.). 1μL of the indicated viruses were administered at a rate of 0.2μL/min bilaterally to the VTA using coordinates from bregma: anterior/posterior: -3.08mm, medial/lateral: ±0.5mm, dorsal/ventral: -4.5mm. Syringes were maintained in position for a minimum of 5 minutes post-infusion prior to removal. Viral incubation was for either 4–5 weeks or 5–6months. Viral expression was confirmed by visualizing midbrain GFP reporters encoded in the viral constructs and/or by RT-qPCR.

### RNA Extraction and RT-qPCR

Bilateral 1.0mm^2^ tissue punches were obtained from 300μm coronal ventral midbrain slices of experimental mice. Punches were collected while visualizing GFP on an inverted fluorescence microscope and RNA was extracted immediately, or following tissue storage at −70°C, using RNAqueous^®^-Micro Kit RNA isolation (Thermo Fisher Scientific). Extracted RNA was reverse transcribed using RETROscript^®^ reverse transcription kit (Thermo Fisher Scientific). Quantitative PCR was performed using the Applied Biosystems^®^ 7500 Real-Time PCR System Machine and software or using the Bio-Rad C1000 Touch Thermal Cycler with CFX96 Real-Time system and software using Taqman^®^ gene expression assays for mouse Rit2 (Mm0172749_mH), TH (Mm00447557_m1), DAT (Mm00438388_m1), DRD2 (Mm00438541_m1), Pitx3 (Mm01194166_g1), Nurr1 (Nr4a2, Mm00443060_m1), Rit1 (Mm00501400_m1), Vps35 (Mm00458167_m1). All Ct values were normalized to internal GAPDH (Mm99999915_g1) expression levels, to determine DCt values. For linear comparisons, data were analyzed by comparing 2^−ΔCt^ values.

### Mouse Behavior

#### Locomotion:

Mouse activity was monitored individually in photobeam activity chambers (San Diego Instruments) as previously described (34). Horizontal, vertical, and fine movements were measured in 5-minute binds for 90 minutes total.

#### Accelerating and Fixed-Speed rotarod:

Mice were habituated to the testing room in home cage for >30min with ambient lighting and the rotarod (UgoBasile 47600) running at 4 RPM*. Accelerating rotarod*: Mice were placed on the rod moving at 4 RPM and rod speed was increased linearly from 4 to 40 RPM over 5 minutes. Trials were terminated and latency determined by either triggering the strike plate during a fall or if the mouse made >1 consecutive passive rotation. *Fixed-speed*: Mice were placed on the rod moving at the indicated speeds (20, 25, 30, 35, 40, 45 RPM) for 60 second trials. Latency to fall was measured or trial was stopped following >1 passive rotation. Two consecutive trials were performed per speed and latencies were averaged per animal.

#### Challenge Balance Beam:

Mice were habituated to the testing room for >30min with overhead lights off and only a single light source placed approximately 1.5 feet over the beam origin illuminated. On day one, mice were trained over 5 trials to traverse a 1.0m, step-wise tapered (widths: 35mm, 25mm, 15mm, 5mm) elevated beam (#80306, Lafayette Neuroscience) at an incline of 15°. A dark box with home-cage bedding was placed at the far end of the beam. On day two, a challenge grid with 1cm × 1cm openings (custom 3D-printed, Thingiverse #4869650) was placed over the beam and mice traversed the beam in 3 independent trials. Traversal initiation and completion were determined by breaking an IR beam at each end of the beam. Traversals were video captured and scored for foot faults and traversal time, averaged over the first two completed trials. Both the experimenter and an independent scorer were double-blinded to the mouse ID.

#### Gait Analysis:

Gait analysis protocol was adapted from Wertman, *et al*. (49). Gait testing apparatus consisted of a 10cm × 36cm runway with 14cm high foamboard walls and a dark box at the opposing end. Fresh, legal-size paper was placed on the benchtop under the runway for each trial. Mouse forepaws and hind-paws were dipped in non-toxic orange and blue tempera paint, respectively, and mice were placed on the paper at the open end of the runway and allowed to traverse to the closed box at the opposing end. Three trials were performed per mouse and stride length, stride width and tow spread were measured for both fore- and hindlimbs. Number of completed trials was also quantified. Experimenters and data analysts were double-blinded to mouse IDs.

#### Grip Strength:

Four-limb grip strength was measured using the Bioseb Grip Strength Test (BIOGS3) equipped with mesh grip grid for mice. Mice were suspended by tail over the mesh and lowered onto it until all 4 paws grasped the mesh. The mouse was then pulled backwards gently on the horizontal plane until it released from the mesh. The maximal force applied was recorded for 3 consecutive trials and averaged.

### Tissue harvesting and immunoblotting

Striata were collected by preparing 300μm coronal sections on a Vibratome as previously described(8,9). Sections were collected through the entire striatum, dorsal and ventral striata were subdissected, and slices encompassing each region were pooled for each independent mouse. Tissue was lysed in RIPA buffer (10mM Tris, pH 7.4; 150mM NaCl; 1.0mM EDTA; 0.1% SDS, 1% Triton X-100, 1% Na deoxycholate) supplemented with protease inhibitors (1.0mM phenylmethylsulfonyl fluoride and 1.0g/mL each leupeptin, aprotinin, and pepstatin) and Phosphatase inhibitor cocktail V (EMD Millipore). Mechanical tissue disruption was also performed by triturating sequentially through a 200μL pipette tip, 22-, and 26- gauge tech tips and solubilized by rotating (30min 4°C). Insoluble material was removed by centrifugation (15min, 18K × g, 4°C). Lysate protein concentrations were determined by BCA protein assay (Thermo Fisher Scientific). Protein samples were denatured in an equal volume of 2x Laemmli sample buffer and were either rotated (30min, RT) for membrane protein immunoblots or boiled (5min) for soluble protein immunoblots. Proteins were resolved by SDS-Page, transferred to nitrocellulose membranes, and the indicated proteins were detected and quantified by immunoblotting with the following antibodies: rat anti-DAT (MAB369, Millipore; 1:2000), rabbit anti-TH (AB152, Millipore, 1:10000), rabbit anti-pSer40 TH (AB5935, Millipore, 1:5000), rabbit anti-αSyn, rabbit anti-pSer129-αSyn, anti-LRRK2, anti-pSer935 LRRK2, mouse anti-actin (Santa Cruz, 1:5000). Secondary antibodies conjugated to horseradish peroxidase were all from Jackson ImmunoResearch and immunoreactive bands were visualized by chemiluminescence using SuperSignal West Dura (Thermo Scientific). Immunoblotting solutinos were prepared in either PBS-T, or TBS-T (137mM NaCl, 2.7mM KCl, 19mM Tris base, ph7.4, 0.1% Tween20) when probing for phosphoproteins. Non-saturating immunoreactive bands were detected using either VersaDoc 5000MP or Chemidoc imaging stations (Bio-Rad) and were quantified using Quantity One software (Bio-Rad). Representative blots shown for a given condition were cropped from the same exposure of the same immunoblot and spliced together for presentation purposes only. Splice margins are indicated with a line. Brightness and contrast settings were identical for all immunoblot images presented.

### Fast-Scan Cyclic Voltammetry

Mice were sacrificed by cervical dislocation and rapid decapitation. Heads were immediately submerged in ice-cold NMDG cutting solution, pH 7.3–7.4 (20mM HEPES, 2.5mM KCl, 1.25mM NaH_2_PO_4_, 30mM NaHCO_3_, 25mM glucose, 0.5mM CaCl_2_·4H_2_O. 10mM MgSO_4_·7H_2_O, 92mM Nmethyl-D-glucamine, 2mM thiourea, 5mM Na^+^-ascorbate, 3mM Na^+^-pyruvate). Brains were removed, glued to the stage of a VT1200S Vibroslicer (Leica) and submerged in ice-cold, oxygenated cutting solution. 300μm slices were prepared and were hemisected along the midline prior to recovering in ACSF (125mM NaCl, 2.5mM KCl, 1.24mM NaH_2_PO_4_, 26mM NaHCO_3_, 11mM glucose, 2.4mM CaCl_2_·4H_2_O,1.2mM MgCl_2_·6H_2_O, pH 7.4) at 31°C for a minimum of 1 hour prior to recording. Hemislices were moved to the recording chamber and were perfused with oxygenated ASCF supplemented with 500μM Na-Ascorbate. Glass pipettes containing a 7μm carbon-fiber microelectrode were prepared and preconditioned in ASCF by applying triangular voltage ramps (−0.4 to +1.2 and back to −0.4 V at 400 V/s), delivered at 60Hz for 1 hour. Recordings were performed at 10Hz. Electrodes were calibrated to a 1μM DA standard prior to recording. Electrodes were positioned in DS and DA transients were electrically evoked with a 250μA rectangular pulse every 2 min, using a concentric bipolar electrode placed ~100μm from the carbon fiber electrode. Data were collected with a 3-electrode headstage, using an EPC10 amplifier (Heka) after low-pass filter at 10 kHz and digitized at 100 kHz, using Patchmaster software (Heka). A stable baseline was achieved after evoking six consecutive DA transients, after which experimental data were collected. Each biological replicate is the average of three evoked DA transients/slice, and a minimum of 3 independent mice were used to gather data from the indicated number of slices in each experiment. Data were analyzed in Igor Pro, using the Wavemetrics FSCV plugin (gift of Veronica Alvarez, NIAAA). Peak amplitudes were measured for each individual DA transient, and tau was calculated as 1/e according to the equation: y = y0 + A^((x-x0)/tau)).^

### Mass Spectrometry

#### Sample Preparation:

Brains were harvested, 1.0mm coronal sections were prepared and bilateral 1.0mm^2^ punches were each taken from dorsal and ventral striata. Each bilateral pair was solubilized in 10μL internal standard solution (200μM 13C_4_-GABA and 1μM 2H_3_-DA in water with 500μM ascorbic acid and 0.1% formic acid) and 50μl ice-cold acetonitrile with 1% formic acid. Samples were vortexed twice for 0.5min with a 1 min incubation on ice between vortexing and were sonicated in an ice-water bath until tissue was completely disrupted. Samples were centrifuged (10 min, 16,000 × g) and supernatants were collected for LC/MS/MS analysis. A standard (STD) solution containing 200 μM GABA, 1 μM dopamine, 500 μM ascorbic acid and 0.1 % formic acid was also prepared.

#### LC/MS/MS:

10μl samples were injectd in triplicate into a Thermo Scientific Ultimate 3000 HPLC system on a SeQuant ZIC-cHILIC column (2.1 × 100 mm, 3 μm) with a ZIC-cHILIC guard column (2.1 × 20 mm, 5 μm), coupled with a Thermo Scientific TSQ Quantiva triple quadrupole mass spectrometer. The mobile phase was water with 0.1% formic acid (A) and acetonitrile (B), and the elution program was as follows: 0 min 25% A, 0.5 min 25% A, 4.5 min 45% A, 5.0 min 70% A, 8.0 min 70% A, 8.1 min 25% A, 12.0 min 25% A at 0.2 mL/min. Ionization was operated in the positive mode with the voltage of 4.2 kV. The parameters were set as follow: sheath gas, 35 Arb, aux gas, 15 Arb, vaporizer temperature, 250 °C, ion transfer tube temperature, 325 °C. Multiple reaction monitoring (MRM) was performed using a cycle time of 0.3 s, CID gas pressure of 1.5 mTorr, Q1 resolution (FWHM) of 0.7 and Q3 resolution (FWHM) of 0.7. The MRM transitions 104.1>87 (GABA), 108.1>91 (^13^C_4_-GABA), 154.1>91 (dopamine) and 157.1>93 (^2^H_3_dopamine) were selected for quantification. All data was integrated and processed in Xcalibur (Version 2.2, Thermo Scientific).

#### Stereological analysis

SNc total and TH+ neurons were quantified as previously described(50). Briefly, mice were perfused and fixed with freshly made 4% paraformaldehyde (PFA) in PBS. Brains were removed immediately and fixed again in 4% PFA followed by equilibration in 30% sucrose in PBS. Fixed brains were imbedded in the OCT-compound media (Sakura) and frozen in liquid nitrogen. 40 μm cryosections were prepared through the midbrain a Leica CM3050s cryostat, and were stored in an antifreeze media containing 30% ethylene glycol, 25% glycerol, and 5% phosphate buffer. For stereology counting, 1 in every 5 sections was selected with a random start and a total of 6 brain slices on average were used for each mouse for IHC labeling for TH, including DAB enhancement, followed by Cresyl violet staining to reveal all neurons. Substantia nigra pars compacta was imaged using a Zeiss Axioplan 2 microscope equipped with a 20X objective, and Stereo Investigator was used to estimate the total number of neurons in the region of interest using the following parameters: frame sizes, 150 X 150 μm; grid sizes, 250 X 250 μm; top guard zone height, 2 μm; and optical dissector height, 8 μm. These parameters yielded a coefficient of error < 10% throughout the analysis. Total cell numbers measured were weighted to section thickness for each mouse and were averaged across each cohort. Investigators performing stereological counting were blinded to mouse identity.

### Statistics

Data analysis was performed with GraphPad Prism software. All data were assessed for normality and nonparametric tests were applied if data distribution was non-Gaussian. Outliers in each data set were identified using either Grubb’s or Rout’s outlier tests, with a or Q values set at 0.05 or 5%, respectively, and were removed from further analysis. Significant differences between two values were determined using either a one-tailed, two-tailed, or paired Student’s t test, as indicated. Differences amongst more than two conditions were determined using one-way or two-way ANOVA, as appropriate, and significant differences among individual values within the group were determined by post-hoc multiple comparison tests, as described for each experiment.

## Data Availability

All data generated or analysed during this study are included in this published article and its supplementary information files.

## References

[1] Hartmann A. (2004) Postmortem studies in Parkinson’s disease. Dialogues Clin Neurosci 6, 281–293 22033507 10.31887/DCNS.2004.6.3/ahartmann PMC3181805

[2] Dickson D. W. (2012) Parkinson’s disease and parkinsonism: neuropathology. Cold Spring Harb Perspect Med 2 10.1101/cshperspect.a009258 PMC3405828 22908195

[3] Pringsheim T., Jette N., Frolkis A., and Steeves T. D. (2014) The prevalence of Parkinson’s disease: a systematic review and meta-analysis. Mov Disord 29, 1583–1590 24976103 10.1002/mds.25945

[4] Lees A. J. (2007) Unresolved issues relating to the shaking palsy on the celebration of James Parkinson’s 250th birthday. Mov Disord 22 Suppl 17, S327–334 18175393 10.1002/mds.21684

[5] Jankovic J. (2008) Parkinson’s disease: clinical features and diagnosis. J Neurol Neurosurg Psychiatry 79, 368–376 18344392 10.1136/jnnp.2007.131045

[6] Tambasco N., Romoli M., and Calabresi P. (2018) Levodopa in Parkinson’s Disease: Current Status and Future Developments. Curr Neuropharmacol 16, 1239–1252 28494719 10.2174/1570159X15666170510143821 PMC6187751

[7] Zhou Q., Li J., Wang H., Yin Y., and Zhou J. (2011) Identification of nigral dopaminergic neuron-enriched genes in adult rats. Neurobiol Aging 32, 313–326 19303663 10.1016/j.neurobiolaging.2009.02.009

[8] Fagan R. R., Kearney P. J., Sweeney C. G., Luethi D., Schoot Uiterkamp F. E., Schicker K., Alejandro B. S., O’Connor L. C., Sitte H. H., and Melikian H. E. (2020) Dopamine transporter trafficking and Rit2 GTPase: Mechanism of action and in vivo impact. J Biol Chem 295, 5229–5244 32132171 10.1074/jbc.RA120.012628 PMC7170531

[9] Kearney P. J., Bolden N. C., Kahuno E., Conklin T. L., Martin G. E., Lubec G., and Melikian H. E. (2023) Presynaptic Gq-coupled receptors drive biphasic dopamine transporter trafficking that modulates dopamine clearance and motor function. J Biol Chem 299, 102900 36640864 10.1016/j.jbc.2023.102900 PMC9943899

[10] Navaroli D. M., Stevens Z. H., Uzelac Z., Gabriel L., King M. J., Lifshitz L. M., Sitte H. H., and Melikian H. E. (2011) The plasma membrane-associated GTPase Rin interacts with the dopamine transporter and is required for protein kinase C-regulated dopamine transporter trafficking. J Neurosci 31, 13758–13770 21957239 10.1523/JNEUROSCI.2649-11.2011 PMC3205929

[11] Hoshino M., and Nakamura S. (2002) The Ras-like small GTP-binding protein Rin is activated by growth factor stimulation. Biochem Biophys Res Commun 295, 651–656 12099688 10.1016/s0006-291x(02)00731-3

[12] Spencer M. L., Shao H., Tucker H. M., and Andres D. A. (2002) Nerve growth factordependent activation of the small GTPase Rin. J Biol Chem 277, 17605–17615 11877426 10.1074/jbc.M111400200

[13] Hoshino M., and Nakamura S. (2003) Small GTPase Rin induces neurite outgrowth through Rac/Cdc42 and calmodulin in PC12 cells. J Cell Biol 163, 1067–1076 14662747 10.1083/jcb.200308070 PMC2173625

[14] Shi G. X., Han J., and Andres D. A. (2005) Rin GTPase couples nerve growth factor signaling to p38 and b-Raf/ERK pathways to promote neuronal differentiation. J Biol Chem 280, 37599–37609 16157584 10.1074/jbc.M507364200

[15] Latourelle J. C., Dumitriu A., Hadzi T. C., Beach T. G., and Myers R. H. (2012) Evaluation of Parkinson disease risk variants as expression-QTLs. PLoS One 7, e46199 23071545 10.1371/journal.pone.0046199 PMC3465315

[16] Pankratz N., Beecham G. W., DeStefano A. L., Dawson T. M., Doheny K. F., Factor S. A., Hamza T. H., Hung A. Y., Hyman B. T., Ivinson A. J., Krainc D., Latourelle J. C., Clark L. N., Marder K., Martin E. R., Mayeux R., Ross O. A., Scherzer C. R., Simon D. K., Tanner C., Vance J. M., Wszolek Z. K., Zabetian C. P., Myers R. H., Payami H., Scott W. K., Foroud T., and Consortium P. G. (2012) Meta-analysis of Parkinson’s disease: identification of a novel locus, RIT2. Ann Neurol 71, 370–384 22451204 10.1002/ana.22687 PMC3354734

[17] Emamalizadeh B., Movafagh A., Akbari M., Kazeminasab S., Fazeli A., Motallebi M., Shahidi G. A., Petramfar P., Mirfakhraie R., and Darvish H. (2014) RIT2, a susceptibility gene for Parkinson’s disease in Iranian population. Neurobiol Aging 35, e27–e28 25146456 10.1016/j.neurobiolaging.2014.07.013

[18] Nalls M. A., Pankratz N., Lill C. M., Do C. B., Hernandez D. G., Saad M., DeStefano A. L., Kara E., Bras J., Sharma M., Schulte C., Keller M. F., Arepalli S., Letson C., Edsall C., Stefansson H., Liu X., Pliner H., Lee J. H., Cheng R., International Parkinson’s Disease Genomics, C., Parkinson’s Study Group Parkinson’s Research: The Organized, G. I., andMe, GenePd, NeuroGenetics Research, C., Hussman Institute of Human, G., Ashkenazi Jewish Dataset, I., Cohorts for, H., Aging Research in Genetic, E., North American Brain Expression, C., United Kingdom Brain Expression, C., Greek Parkinson’s Disease, C., Alzheimer Genetic Analysis, G., Ikram M. A., Ioannidis J. P., Hadjigeorgiou G. M., Bis J. C., Martinez M., Perlmutter J. S., Goate A., Marder K., Fiske B., Sutherland M., Xiromerisiou G., Myers R. H., Clark L. N., Stefansson K., Hardy J. A., Heutink P., Chen H., Wood N. W., Houlden H., Payami H., Brice A., Scott W. K., Gasser T., Bertram L., Eriksson N., Foroud T., and Singleton A. B. (2014) Large-scale meta-analysis of genome-wide association data identifies six new risk loci for Parkinson’s disease. Nat Genet 46, 989–993 25064009 10.1038/ng.3043 PMC4146673

[19] Lu Y., Liu W., Tan K., Peng J., Zhu Y., and Wang X. (2015) Genetic association of RIT2 rs12456492 polymorphism and Parkinson’s disease susceptibility in Asian populations: a meta-analysis. Sci Rep 5, 13805 26334395 10.1038/srep13805 PMC4558715

[20] Liu Z. H., Guo J. F., Wang Y. Q., Li K., Sun Q. Y., Xu Q., Yan X. X., Xu C. S., and Tang B. S. (2015) Assessment of RIT2 rs12456492 association with Parkinson’s disease in Mainland China. Neurobiol Aging 36, 1600 e1609–1611 10.1016/j.neurobiolaging.2014.12.012 25559334

[21] Wang J. Y., Gong M. Y., Ye Y. L., Ye J. M., Lin G. L., Zhuang Q. Q., Zhang X., and Zhu J. H. (2015) The RIT2 and STX1B polymorphisms are associated with Parkinson’s disease. Parkinsonism Relat Disord 21, 300–302 25534083 10.1016/j.parkreldis.2014.12.006

[22] Zhang X., Niu M., Li H., and Xie A. (2015) RIT2 rs12456492 polymorphism and the risk of Parkinson’s disease: A meta-analysis. Neurosci Lett 602, 167–171 26188085 10.1016/j.neulet.2015.07.004

[23] Chan G., White C. C., Winn P. A., Cimpean M., Replogle J. M., Glick L. R., Cuerdon N. E., Ryan K. J., Johnson K. A., Schneider J. A., Bennett D. A., Chibnik L. B., Sperling R. A., De Jager P. L., and Bradshaw E. M. (2016) Trans-pQTL study identifies immune crosstalk between Parkinson and Alzheimer loci. Neurol Genet 2, e90 27504496 10.1212/NXG.0000000000000090 PMC4962525

[24] Emamalizadeh B., Jamshidi J., Movafagh A., Ohadi M., Khaniani M. S., Kazeminasab S., Biglarian A., Taghavi S., Motallebi M., Fazeli A., Ahmadifard A., Shahidi G. A., Petramfar P., Shahmohammadibeni N., Dadkhah T., Khademi E., Tafakhori A., Khaligh A., Safaralizadeh T., Kowsari A., Mirabzadeh A., Zarneh A. E. S., Khorrami M., Shokraeian P., Banavandi M. J. S., Lima B. S., Andarva M., Alehabib E., Atakhorrami M., and Darvish H. (2017) RIT2 Polymorphisms: Is There a Differential Association? Mol Neurobiol 54, 2234–2240 26941103 10.1007/s12035-016-9815-4

[25] Chang D., Nalls M. A., Hallgrimsdottir I. B., Hunkapiller J., van der Brug M., Cai F., International Parkinson’s Disease Genomics, C., andMe Research, T., Kerchner G. A., Ayalon G., Bingol B., Sheng M., Hinds D., Behrens T. W., Singleton A. B., Bhangale T. R., and Graham R. R. (2017) A meta-analysis of genome-wide association studies identifies 17 new Parkinson’s disease risk loci. Nat Genet 49, 1511–1516 28892059 10.1038/ng.3955 PMC5812477

[26] Daneshmandpour Y., Darvish H., and Emamalizadeh B. (2018) RIT2: responsible and susceptible gene for neurological and psychiatric disorders. Mol Genet Genomics 293, 785–792 29860660 10.1007/s00438-018-1451-4

[27] Glessner J. T., Reilly M. P., Kim C. E., Takahashi N., Albano A., Hou C., Bradfield J. P., Zhang H., Sleiman P. M., Flory J. H., Imielinski M., Frackelton E. C., Chiavacci R., Thomas K. A., Garris M., Otieno F. G., Davidson M., Weiser M., Reichenberg A., Davis K. L., Friedman J. I., Cappola T. P., Margulies K. B., Rader D. J., Grant S. F., Buxbaum J. D., Gur R. E., and Hakonarson H. (2010) Strong synaptic transmission impact by copy number variations in schizophrenia. Proc Natl Acad Sci U S A 107, 10584–10589 20489179 10.1073/pnas.1000274107 PMC2890845

[28] Liu H., Talalay P., and Fahey J. W. (2016) Biomarker-Guided Strategy for Treatment of Autism Spectrum Disorder (ASD). CNS Neurol Disord Drug Targets 15, 602–613 27071792 10.2174/1871527315666160413120414

[29] Hamedani S. Y., Gharesouran J., Noroozi R., Sayad A., Omrani M. D., Mir A., Afjeh S. S. A., Toghi M., Manoochehrabadi S., Ghafouri-Fard S., and Taheri M. (2017) Ras-like without CAAX 2 (RIT2): a susceptibility gene for autism spectrum disorder. Metab Brain Dis 32, 751–755 28190241 10.1007/s11011-017-9969-4

[30] Bouquillon S., Andrieux J., Landais E., Duban-Bedu B., Boidein F., Lenne B., Vallee L., Leal T., Doco-Fenzy M., and Delobel B. (2011) A 5.3Mb deletion in chromosome 18q12.3 as the smallest region of overlap in two patients with expressive speech delay. Eur J Med Genet 54, 194–197 21145994 10.1016/j.ejmg.2010.11.009

[31] Bossers K., Meerhoff G., Balesar R., van Dongen J. W., Kruse C. G., Swaab D. F., and Verhaagen J. (2009) Analysis of gene expression in Parkinson’s disease: possible involvement of neurotrophic support and axon guidance in dopaminergic cell death. Brain Pathol 19, 91–107 18462474 10.1111/j.1750-3639.2008.00171.x PMC8094761

[32] Wang Q., Wang M., Choi I., Ho L., Farrell K., Beaumont K. G., Sebra R., Crary J. F., Davis D. A., Sun X., Zhang B., and Yue Z. (2022) Single-cell transcriptomic atlas of the human substantia nigra in Parkinson’s disease. bioRxiv, 2022.2003.2025.485846

[33] Obergasteiger J., Castonguay A. M., Pizzi S., Magnabosco S., Frapporti G., Lobbestael E., Baekelandt V., Hicks A. A., Pramstaller P. P., Gravel C., Corti C., Levesque M., and Volta M. (2023) The small GTPase Rit2 modulates LRRK2 kinase activity, is required for lysosomal function and protects against alpha-synuclein neuropathology. NPJ Parkinsons Dis 9, 44 36973269 10.1038/s41531-023-00484-2 PMC10042831

[34] Sweeney C. G., Kearney P. J., Fagan R. R., Smith L. A., Bolden N. C., Zhao-Shea R., Rivera I. V., Kolpakova J., Xie J., Gao G., Tapper A. R., Martin G. E., and Melikian H. E. (2020) Conditional, inducible gene silencing in dopamine neurons reveals a sex-specific role for Rit2 GTPase in acute cocaine response and striatal function. Neuropsychopharmacology 45, 384–393 31277075 10.1038/s41386-019-0457-x PMC6901441

[35] Ford C. P. (2014) The role of D2-autoreceptors in regulating dopamine neuron activity and transmission. Neuroscience 282, 13–22 24463000 10.1016/j.neuroscience.2014.01.025 PMC4108583

[36] Sato H., Kato T., and Arawaka S. (2013) The role of Ser129 phosphorylation of alpha-synuclein in neurodegeneration of Parkinson’s disease: a review of in vivo models. Rev Neurosci 24, 11–123 10.1515/revneuro-2012-0071 23314528

[37] Casiraghi A., Longhena F., Faustini G., Ribaudo G., Suigo L., Camacho-Hernandez G. A., Bono F., Brembati V., Newman A. H., Gianoncelli A., Straniero V., Bellucci A., and Valoti E. (2022) Methylphenidate Analogues as a New Class of Potential DiseaseModifying Agents for Parkinson’s Disease: Evidence from Cell Models and Alpha-Synuclein Transgenic Mice. Pharmaceutics 14 10.3390/pharmaceutics14081595 PMC9414221 36015221

[38] Lou J. S. (2015) Fatigue in Parkinson’s disease and potential interventions. NeuroRehabilitation 37, 25–34 26409691 10.3233/NRE-151238

[39] Eshleman A. J., Carmolli M., Cumbay M., Martens C. R., Neve K. A., and Janowsky A. (1999) Characteristics of drug interactions with recombinant biogenic amine transporters expressed in the same cell type. J Pharmacol Exp Ther 289, 877–885 10215666

[40] Fahn S. (2008) The history of dopamine and levodopa in the treatment of Parkinson’s disease. Mov Disord 23 Suppl 3, S497–508 18781671 10.1002/mds.22028

[41] Augustin S. M., Loewinger G. C., O’Neal T. J., Kravitz A. V., and Lovinger D. M. (2020) Dopamine D2 receptor signaling on iMSNs is required for initiation and vigor of learned actions. Neuropsychopharmacology 45, 2087–2097 32811899 10.1038/s41386-020-00799-1 PMC7547091

[42] Lemos J. C., Friend D. M., Kaplan A. R., Shin J. H., Rubinstein M., Kravitz A. V., and Alvarez V. A. (2016) Enhanced GABA Transmission Drives Bradykinesia Following Loss of Dopamine D2 Receptor Signaling. Neuron 90, 824–838 27196975 10.1016/j.neuron.2016.04.040 PMC4882167

[43] Roberts H. C., Syddall H. E., Butchart J. W., Stack E. L., Cooper C., and Sayer A. A. (2015) The Association of Grip Strength With Severity and Duration of Parkinson’s: A Cross-Sectional Study. Neurorehabil Neural Repair 29, 889–896 25653226 10.1177/1545968315570324

[44] Jeyasingham R. A., Baird A. L., Meldrum A., and Dunnett S. B. (2001) Differential effects of unilateral striatal and nigrostriatal lesions on grip strength, skilled paw reaching and drug-induced rotation in the rat. Brain Res Bull 55, 541–548 11543955 10.1016/s0361-9230(01)00557-3

[45] Decressac M., Volakakis N., Bjorklund A., and Perlmann T. (2013) NURR1 in Parkinson disease--from pathogenesis to therapeutic potential. Nat Rev Neurol 9, 629–636 24126627 10.1038/nrneurol.2013.209

[46] Kadkhodaei B., Alvarsson A., Schintu N., Ramskold D., Volakakis N., Joodmardi E., Yoshitake T., Kehr J., Decressac M., Bjorklund A., Sandberg R., Svenningsson P., and Perlmann T. (2013) Transcription factor Nurr1 maintains fiber integrity and nuclearencoded mitochondrial gene expression in dopamine neurons. Proc Natl Acad Sci U S A 110, 2360–2365 23341612 10.1073/pnas.1221077110 PMC3568335

[47] Jia C., Qi H., Cheng C., Wu X., Yang Z., Cai H., Chen S., and Le W. (2020) alpha-Synuclein Negatively Regulates Nurr1 Expression Through NF-kappaB-Related Mechanism. Front Mol Neurosci 13, 64 32477062 10.3389/fnmol.2020.00064 PMC7235291

[48] Burger C., Nguyen F. N., Deng J., and Mandel R. J. (2005) Systemic mannitol-induced hyperosmolality amplifies rAAV2-mediated striatal transduction to a greater extent than local co-infusion. Mol Ther 11, 327–331 15668145 10.1016/j.ymthe.2004.08.031

[49] Wertman V., Gromova A., La Spada A. R., and Cortes C. J. (2019) Low-Cost Gait Analysis for Behavioral Phenotyping of Mouse Models of Neuromuscular Disease. J Vis Exp 10.3791/59878 PMC7553151 31380846

[50] Wang Q., Zhang Y., Wang M., Song W.-M., Shen Q., McKenzie A., Choi I., Zhou X., Pan P.-Y., Yue Z., and Zhang B. (2019) The landscape of multiscale transcriptomic networks and key regulators in Parkinson’s disease. Nature Communications 10, 5234 10.1038/s41467-019-13144-y PMC6868244 31748532

